# Prognostic Significance of Concurrent Hypovascular and Hypervascular Nodules in Patients with Hepatocellular Carcinoma

**DOI:** 10.1371/journal.pone.0163119

**Published:** 2016-09-20

**Authors:** Sadahisa Ogasawara, Tetsuhiro Chiba, Tenyu Motoyama, Naoya Kanogawa, Tomoko Saito, Yusuke Shinozaki, Eiichiro Suzuki, Yoshihiko Ooka, Akinobu Tawada, Hideyuki Kato, Shinichiro Okabe, Fumihiko Kanai, Masaharu Yoshikawa, Osamu Yokosuka

**Affiliations:** 1 Department of Gastroenterology and Nephrology, Graduate School of Medicine, Chiba University, Chiba, Japan; 2 Department of Radiology, Chiba University Hospital, Chiba, Japan; Kanazawa Daigaku, JAPAN

## Abstract

**Background:**

Hypovascular nodules often occur together with hypervascular hepatocellular carcinoma (HCC). However, it remains controversial whether hypovascular nodules associated with hypervascular HCC have any prognostic value. This study evaluated the prognostic impact of hypovascular nodules co-existing with hypervascular HCC as diagnosed by computed tomography during arterial portography (CTAP) and computed tomography during hepatic arteriography (CTHA), which can sensitively capture the dynamic changes in blood flow through the portal vein and hepatic artery in patients with early stage HCC.

**Methods:**

A total of 152 patients with hypervascular HCC (≤ 30 mm, ≤ 3 nodules), who underwent initial local ablation, were analyzed retrospectively. All patients received CTAP and CTHA prior to treatment. Overall survival (OS) was compared among group A (hypervascular HCC only, 107 patients) and group B (hypovascular nodules and hypervascular HCC, 45 patients).

**Results:**

Among all hypovascular nodules, 81% (46 of 57) developed hypervascularization within the follow-up period. The 1- and 2-year hypervascularization rates were 17% and 51%, respectively. OS was significantly longer for group A than for group B (*P* < 0.001). A Cox proportional-hazards model identified the presence of hypovascular nodules concurrent with hypervascular HCC as an independent poor prognostic factor.

**Conclusion:**

The prognosis of hypervascular HCC patients with hypovascular nodules detected during CTAP and CTHA is poor. Clinical HCC categories seem to be dissimilar between patients with and without hypovascular nodules.

## Introduction

Hepatocellular carcinoma (HCC) is the sixth most common cancer and the third leading cause of cancer-related deaths worldwide [1]. HCC develops through a multistep carcinogenic process from a dysplastic nodule (DN), to well-differentiated HCC, and finally to overt hypervascular HCC (moderate to poorly differentiated HCC) [2–5]. There is a well-known correlation between the hemodynamics of HCC and its physiology. Angiogenesis, with sinusoidal capillarization and unpaired arteries, increases during multistep progression from a DN to overt hypervascular HCC. The intranodular portal blood supply is decreased in accordance with the progression of angiogenesis. Meanwhile, the intranodular arterial blood supply first decreases during the early stages of carcinogenesis but subsequently increases in parallel with the progressive increase in the grade of malignancy. This progression has mainly been studied through computed tomography (CT) during arterial portography (CTAP) and CT during hepatic arteriography (CTHA) [6–8] because this imaging can sensitively capture the dynamic changes in blood flow through the portal vein and hepatic artery. Most hypovascular nodules, which are defined as having decreased portal perfusion from that of the background liver by CTAP imaging and no increase in arterial perfusion, are DNs or well-differentiated HCCs.

Hypovascular nodules do not only appear exclusively but also concur with hypervascular HCC. These two appearance patterns are considered to be separate clinical conditions. Although recent publications have indicated the prognosis of hypovascular nodules that appear alone [9, 10], few reports are available on hypovascular nodules coincident with hypervascular HCC. Previously, we analyzed patients with intermediate stage HCC and demonstrated that the number of lesions, the Child–Pugh score, and HCV-RNA positivity were served as independent prognostic factors [11]. In view of management of early stage HCC, it is of importance to clarify the prognostic impact of hypovascular nodules with or without hypervascular HCC. The aim of this study was to evaluate the prognostic impact of hypovascular nodules that occur with hypervascular HCC as diagnosed by CTAP and CTHA in patients with early stage HCC.

## Patients and Methods

### Ethics statement

This study was approved by the Research Ethics Committees of Graduate School of Medicine, Chiba University (approval number 2,058). According to the policy of our institution, all of patients including in this study obtained informed consents of examinations and treatments. Patient records/information were anonymized and de-identified prior to analysis.

### Patient

Medical records were retrieved for consecutive patients with HCC treated at our institution. Patients were selected using the following inclusion criteria assessed at their initial local ablation: (1) pathological HCC diagnosis of hypervascular nodules (at least one nodule); (2) ≤ 3 hypervascular HCC lesions that were ≤ 30 mm (without macrovascular invasion or extrahepatic metastasis); (3) Child–Pugh A or B; and (4) CTAP/CTHA performed before initiation of local therapy. Patients meeting these requirements were divided into two groups: hypervascular HCC only (no hypovascular lesions) (group A) and hypovascular nodules co-existing with hypervascular HCC (group B). All patients provided written informed consent for their examination that included CTAP/CTHA and treatment according to the standard practice at our institution.

### CTAP/CTHA protocol

CTAP/CTHA was performed as described previously [12]. Briefly, an IVR-CT system (Infinix Active; Toshiba Medical Systems) with a digital subtraction angiography system (CAS-8000 V/DFP-2000A; Toshiba Medical Systems) and a 4-detector MDCT scanner (Aquilion; Toshiba Medical Systems) was used. At first, 4-Fr angiographic catheter was positioned in the proximal superior mesenteric artery using the Seldinger technique. After the catheter placement, 10 μmol of alprostadil (Palux; Taisho, Tokyo, Japan) was administrated into the SMA to increase the portal blood flow. Subsequently, 30 ml of iohexol (Omnipaque 300; Daiichi-Sankyo Co. Ltd., Tokyo, Japan) was injected at a rate of 3.0 ml/s to obtain CTAP images.

We then placed a catheter tip in the common or proper hepatic artery using the coaxial 2.7-Fr microcatheter system and then injected 18 ml of iohexol at a rate of 2.0 ml/s for CTHA. Two- and three-phase imaging was conducted for CTAP and CTHA, respectively. The scan delay times after starting the injection were 20 and 35 s for CTAP and 3, 18, and 60 s for CTHA.

### Imaging analysis

**Fig 1:**
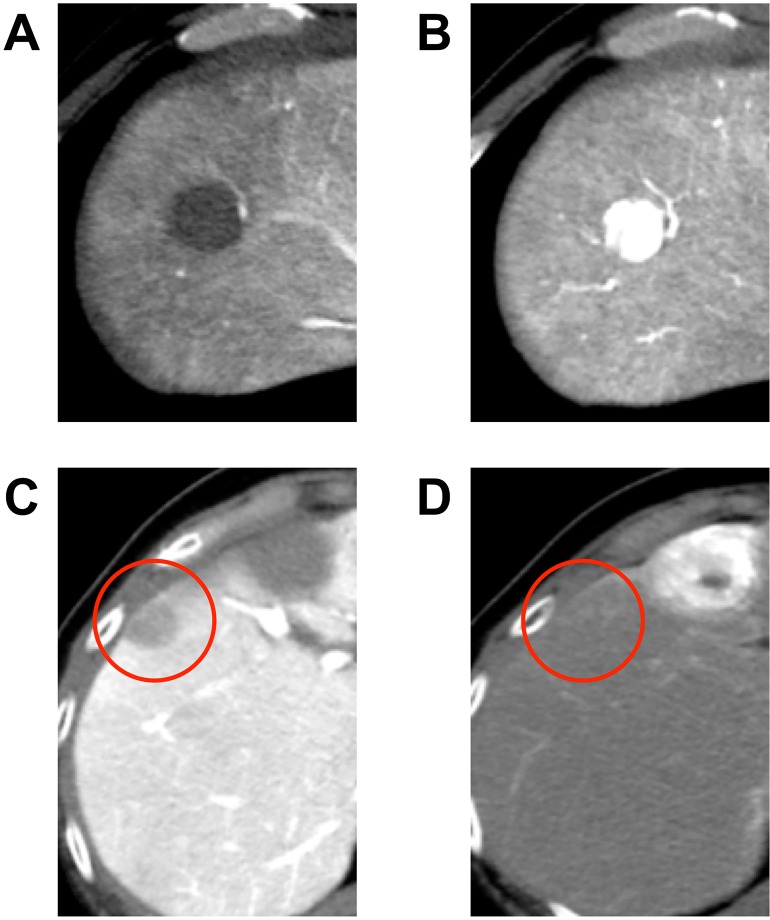
A typical nodule of hypervascular hepatocellular carcinoma (HCC) (A: CTAP, B: CTHA) and a hypovascular nodule (red circle, C: CTAP, D: CTHA). CTAP, computed tomography during arterial portography; CTHA, computed tomography during hepatic arteriography.

Pretreatment CTAP/CTHA results were reviewed by two hepatologists who specialized in HCC (TM and YO). The nodules were retrospectively classified into hypervascular and hypovascular nodules (Fig 1). We defined hypervascular nodules as decreased or no perfusion at CTAP and increased perfusion at CTHA. The nodules with an internal hypervascular focus on CTHA were also classified as hypervascular nodules. Hypovascular nodules showed either equivalent or decreased portal perfusion from that of the background liver at CTAP imaging and no increased arterial perfusion at CTHA [8]. Both hypervascular and hypovascular nodules were detected as lesions in CTAP/CTHA.

### Initial local ablation, follow-up and hypervascularization

Radiofrequency ablation (RFA) was the first-choice local ablation therapy in our institution after it was approved in April 2004 by the Ministry of Health, Labour and Welfare of Japan. Percutaneous ethanol injection (PEI) was performed in cases before RFA was approved, and in patients with no indication for RFA. During follow-up, tumor markers, including alfa fetoprotein (AFP) and des-γ-carboxy prothrombin, were measured every 1–2 months, ultrasonography was performed every 2–4 months, and dynamic computed tomography/magnetic resonance imaging (CT/MRI) was performed every 4–6 months. In cases with suspected new HCC development or hypervascularization of previously hypovascular nodules, at least two radiological imaging examinations, including dynamic CT, dynamic MRI, and/or CTAP/CTHA, were performed to confirm diagnoses. Hypervascularization was defined as a nodule that showed hypervascularity in arterial-phase images and hypovascularity in portal venous or equilibrium-phase images of dynamic CT or dynamic MRI. In addition, we classified nodules as hypervascular when they showed the CTAP/CTHA hypervascular criteria defined above.

### Statistical analysis

Time-to-event data were estimated by Kaplan–Meier plots and calculation of the median (95% confidence interval [95% CI]). Demographic and clinical characteristics were compared using the chi-squared test or Fisher’s exact test, as appropriate. Time to hypervascularization was defined as the period from initial treatment until diagnosis of hypervascularization. The censoring date was defined as the last radiological assessment date.

Recurrence-free survival (RFS) was measured from the date of initial treatment until the date of initial recurrence, or death. The censoring date was defined as the date of last follow-up. Local recurrence was excluded from the analysis. Overall survival (OS) was measured from the date of initial ablation until the date of death. The censoring date was defined as the date of last follow-up. Univariate and multivariate Cox proportional-hazard regression models were used to estimate the hazard ratios for risk factors in relation to OS. A probability value of < 0.05 was considered statistically significant. All statistical analyses were performed using SPSS statistical software (Version 22; SPSS-IBM, Chicago, IL, USA).

## Results

### Patient baseline characteristics

**Fig 2:**
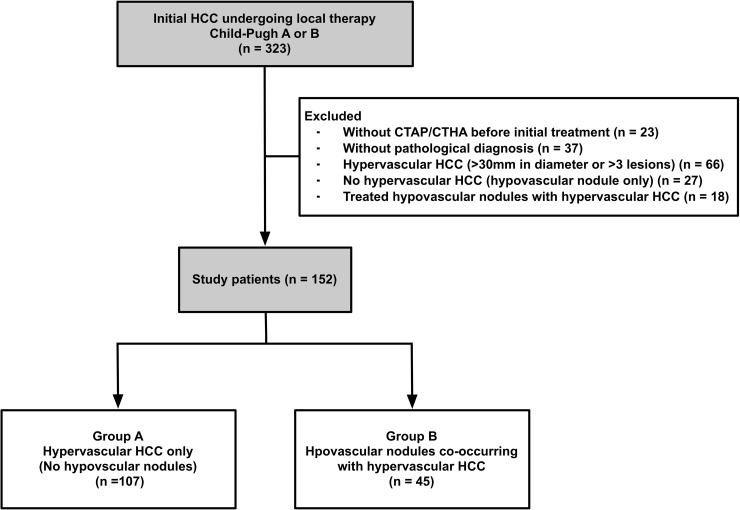
Study flow.

Between April 2000 and March 2010, 323 consecutive patients with HCC who received initial local therapy were identified for potential inclusion in this study (Fig 2). Among the remaining 152 patients who were included in this study, 107 patients had only hypervascular HCC (group A) and 45 patients had hypovascular nodules concurrent with hypervascular HCC (group B). Patient demographics and characteristics are presented in Table 1. There were no significant differences in the baseline characteristics between groups, although there were fewer Child-Pugh A patients, hepatitis B surface antigen (HBs-Ag) positive patients and single nodule and ≤ 20 mm patients in group B compared with group A.

**Table 1:** Baseline demographic data of the study patients.

	Hypervascular HCC only (Group A)	Hypovascular nodulesco-existing with hypervascular HCC (Group B)	*P*
**Patients, n**	107	45	
**Gender,** n (%)			
Male	79 (74)	31 (69)	0.555
Female	28 (26)	14 (31)	
**Age** in years, n (%)			
≤ 67	58 (54)	20 (44)	0.291
> 67	49 (46)	25 (56)	
Median (range)	67 (41–80)	69 (45–85)	
**Child–Pugh class**, n (%)			
A	87 (81)	32 (71)	0.197
B	20 (19)	13 (29)	
**HBs-Ag positive,** n (%)			
Absent	94 (88)	44 (98)	0.066
Present	13 (12)	1 (2)	
**HCV-Ab positive,** n (%)			
Absent	20 (19)	6 (13)	0.488
Present	87 (81)	39 (87)	
**Single hypervascular HCC, ≤ 20 mm,** n (%)			
Absent	56 (52)	28 (62)	0.288
Present	51 (48)	17 (38)	
**AFP, ng/ml,** n (%)			
≤ 100	85 (79)	39 (87)	0.364
> 100	22 (21)	6 (13)	
Median (range)	18.4 (1.8–1997.2)	15.0 (3.0–1315.0)	
**Initial local therapy,** n (%)			
RFA	62 (58)	27 (60)	0.858
PEI	45 (42)	18 (40)	

Abbreviations: HBs-Ag, hepatitis B surface antigen; HCV-Ab, hepatitis C virus antibody; HCC, hepatocellular carcinoma; AFP, alfa fetoprotein; RFA, radiofrequency ablation; PEI, percutaneous ethanol injection.

### Hypovascular lesions and time to progress to hypervascularization

**Fig 3:**
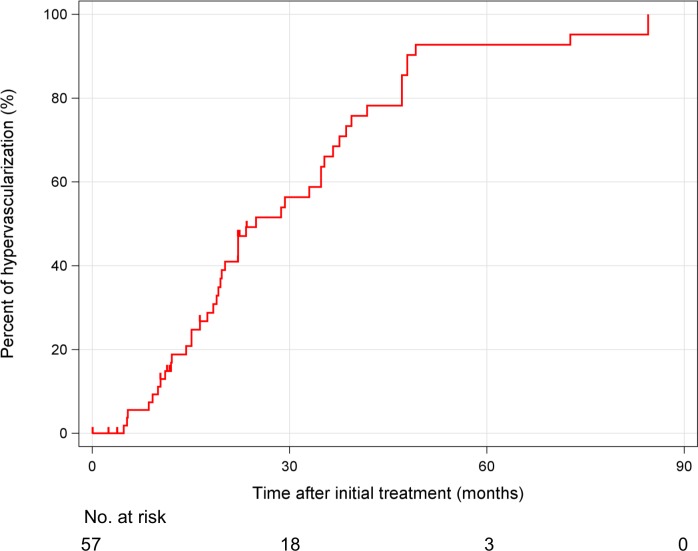
Kaplan–Meier curve for the cumulative hypervascularization rate of hypovascular nodules.

The median size of the 57 hypovascular nodules was 10 mm (range, 5–30 mm) and 81% (46 nodules) became hypervascularized within the follow-up period. The 1- and 2-year hypervascularization rates were 17% and 51%, respectively. Median time for progression to hypervascularization was 24.9 months (95% CI: 16.5–33.2 months; Fig 3). Of 45 patients who had hypovascular nodules, there were no significant differences of time to hypervascularization according to size and number of hypovascular nodules [S1 Fig ≤ 10 mm: 19.5 months (95% CI: 13.3–25.7 months), > 10 mm: 22.2 months (95% CI: 14.4–30.0 months), *P* = 0.791, single: 19.7 months (95% CI: 17.4–22.0), multiple: 24.9 months (95% CI: 18.3–31.5 months, *P* = 0.258)]. Similarly, there were no significant differences of OS according to size and number of hypovascular nodules [S2 Fig ≤ 10 mm: 38.6 months (95% CI: 29.7–47.5 months), > 10 mm: 52.1 months (95% CI: 46.8–57.4 months), *P* = 0.935, single: 48.5 months (95% CI: 34.6–62.4), multiple: 52.1 months (95% CI: 22.9–81.3 months, *P* = 0.198)].

### Impact of recurrence and prognosis of hypovascular nodules

**Fig 4:**
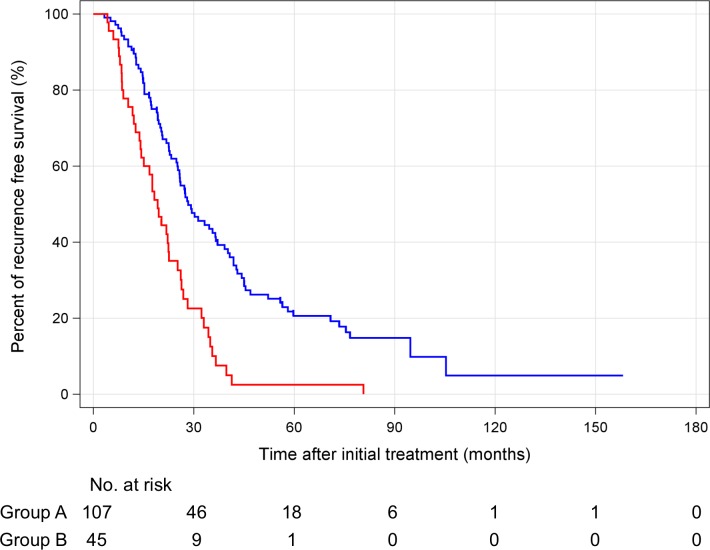
Kaplan-Meier curve recurrence free survival curves for hypervascular hepatocellular carcinoma only (group A, blue line) and hypovascular nodules co-existing with hypervascular hepatocellular carcinoma (group B, red line).

**Fig 5:**
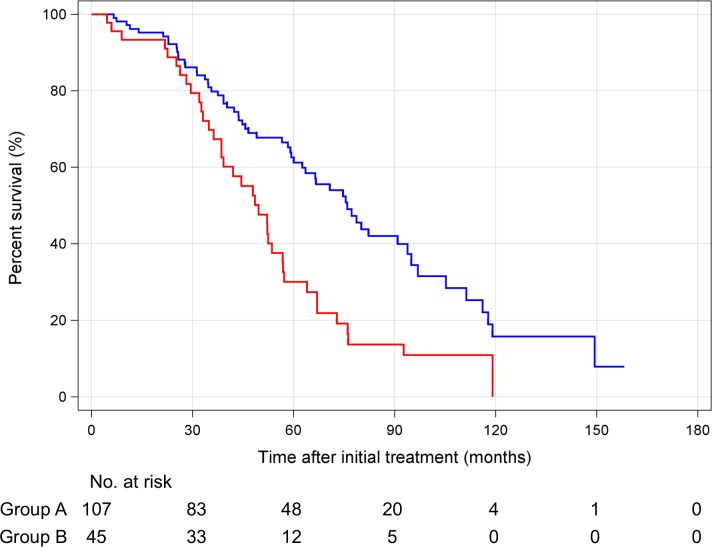
Kaplan–Meier survival curves for hypervascular hepatocellular carcinoma only (group A, blue line) and hypovascular nodules co-existing with hypervascular hepatocellular carcinoma (group B, red line).

According to the Response Evaluation Criteria in Cancer of the Liver (RECICL) [13], 92 patients (86%) and 42 patients (93%) achieved TE4 at initial ablation in groups A and B, respectively (*P* = 0.275). By the end of the study, HCC recurrence occurred in 72 patients (67%) in group A and 36 patients (80%) in group B (S1 Table). There were no significant differences in stage, Child-Pugh class at the time of initial recurrence, and treatment of initial recurrence in both groups. The treatments of clinical course of HCC are shown in S2 Table. Of 13 patients who were HBs-Ag positive in group A, 9 patients received nucleos(t)ide analogue (NA). On the other hand, 1 of 1 patient who were HBs-Ag positive in group B received NA. There were no significant differences in number of treatments, local ablation, and TACE between groups A and B. In this study, there were no patients who received liver transplantation during their clinical course of HCC. Ninety-five patients died during the observation period. The cause of death was not significantly different between groups A and B. The RFS of groups A and B was 28.3 months (95% CI: 22.0–34.7 months) and 19.2 months (95% CI: 15.4–22.9 months), respectively (Fig 4). The OS of groups A and B was 75.9 months (95% CI: 63.3–88.5 months) and 49.6 months (95% CI: 40.2–58.2 months), respectively (Fig 5). The median RFS and OS were significantly longer in group A (RFS: *P* < 0.001, OS: *P* < 0.001). In the multivariate analysis, hypovascular nodules that occurred with hypervascular HCC were an independent poor prognosis factor (Table 2; univariate analysis is shown in S3 Table).

**Table 2:** Multivariate survival analyses based on the contribution of hypovascular nodule prognosis.

Variables	Multivariate analysis		*p*
	Hazard ratio	95% CI	
**Hypovasclar nodules**			
Absent	Reference		
Present	2.072	1.364–3.148	0.001

## Discussion

We have shown that most of the hypovascular nodules diagnosed using CTAP/CTHA developed into overt HCC and patients with both hypovascular nodules and hypervascular HCC had a poorer prognosis than patients with only hypervascular HCC. These results indicated that clinical HCC categories are dissimilar between patients with and without hypovascular nodules.

The majority of the hypovascular nodules in the present study could be classified as DNs or well-differentiated HCCs, although they may not have been diagnosed correctly. A histological diagnosis should be obtained with a biopsy under ultrasound guidance. Several guidelines recommend that typical hypervascular nodules can be diagnosed as HCC without biopsy and that detection of atypical hepatic nodules, such as hypovascular nodules requires a biopsy to prove HCC [14–16]. If patients have hypovascular nodules alone (without hypervascular HCC), biopsy should be considered. However, if patients have hypovascular nodules concurrent with hypervascular HCC, it is not always possible to perform a biopsy of the hypovascular nodules to guide treatment. Major guidelines might not expect to find hypovascular nodules occurring together with hypervascular HCC.

Recently, gadolinium-ethoxybenzyl-diethylenetriamine pentaacetic acid (Gd-EOB-DTPA)-enhanced MRI has been spread in filed practice [16]. In the hepatobiliary phase of Gd-EOB-DTPA-enhanced MRI, hepatic lesions that lack normally functioning hepatocytes are imaged as an absence of hepatocyte-selective enhancement compared with normal parenchyma [17, 18]. Gd-EOB-DTPA-enhanced MRI can detect non-hypervascular hepatic nodules as hypointense nodules during the hepatobiliary phase. Several studies have reported a rate of hypervascularization of non-hypervascular hypointense nodules (Inoue et al.: 1-year = 14.9%, 2-year = 45.8%; Hyodo et al.: 1-year = 25%, 2-year = 46%) [19, 20]. These results were similar to hypervascularization of hypovascular nodules in our study, although there was the limitation that hypervascularization was diagnosed not with only CTAP/CTHA but also other modalities such as dynamic CT or dynamic MRI. Toyoda et al found that non-hypervascular hypointense nodules are a risk factor for recurrence of HCC after hepatectomy, mainly due to multicentric recurrence [21]. Furthermore, patients with non-hypervascular hypointense nodules are at a high risk for HCC development at any site in the liver [22]. Taken together, the livers of patients with DNs or well-differentiated HCC co-existing with overt HCC may have a higher potential for carcinogenesis, or undetectable precursor lesions may be present at other sites in the liver. In the present study, RFS was significantly longer in patients with only hypervascular HCC compared with in patients with both hypovascular nodules and hypervascular HCC. These results may be explained by both the high incidence of hypervascularization of hypovascular nodules, and the high potential of carcinogenesis of the liver that existed with hypovascular nodules. Although statuses of initial recurrence were similar between patients with only hypervascular HCC and patients with both hypovascular nodule and hypervascular HCC, differences in RFS seemed to correlate with differences in OS.

Since CTAP/CTHA is an invasive examination for detecting HCC, it has been replaced with Gd-EOB-DTPA-enhanced MRI [12, 16]. To date, CTAP/CTHA is rarely performed for diagnosis of hypovascular nodules in clinical practice. However, Gd-EOB-DTPA-enhanced MRI has been used since the last decade of the 2000s. Currently, patients with early-stage HCC who receive local ablation extend survival by more than 60 months [23]. Thus, the timeframe of approval of Gd-EOB-DTPA-enhanced MRI to the present may not allow a sufficiently long observation period for analyzing impact of survival of patients who have non-hypervascular hypointense nodules co-existing with hypervascular HCC. Our results document the importance that patients with both hypovascular nodules and hypervascular HCC had a poorer prognosis than patients with only hypervascular HCC. We believe that non-hypervascular hypointense nodules co-existing with hypervascular HCC on Gd-EOB-DTPA-enhanced MRI will have a poor prognosis, similar to the patients identified in our study, and these results should be indicated in the near future.

Hypovascular nodules are often seen in practice. However, it is still controversial whether treating hypovascular nodules that occur with hypervascular HCC has any survival benefit, especially in early stage HCC patients who have an indication of curative therapy. Further large-scale and longer observation-period studies of hypovascular nodules detected by Gd-EOB-DTPA-enhanced MRI are warranted.

In conclusion, hypovascular nodules detected by CTAP/CTHA progress to hypervascular HCC at a high rate, and the patients who had these hypovascular nodules concurrent with hypervascular HCC had a much poorer prognosis. The presence of hypovascular nodules seems to be taken into consideration of the management of early stage HCC.

## Supporting Information

S1 Fig Kaplan-Meier curve of time to hypervascularization for size [A: ≤10 mm (dashed line) vs. > 10 mm (solid line)] and [B: single (dashed line) vs. multiple (solid line)] of hypovascular nodules in patients who had hypovascular nodules.
(TIF)

S2 Fig Kaplan-Meier survival curve for size [A: ≤10 mm (dashed line) vs. > 10 mm (solid line)] and [B: single (dashed line) vs. multiple (solid line)] of hypovascular nodules in patients who had hypovascular nodules.
(TIF)

S1 Table Characteristics of initial recurrence. (PDF)

S2 Table Treatment of clinical course of hepatocellular carcinoma. (PDF)

S3 Table Univariate analysis based on the contribution of hypovascular nodule prognosis. (PDF)

S4 Table Clinical data of the study participants (hypervascular hepatocellular carcinoma only patients: group A). (XLSX)

S5 Table Clinical data of the study participants (both hypervascular hepatocellular carcinoma and hypovascular nodules patients: group B). (XLSX)

S6 Table Clinical data of the study participants (hypovascular nodules). (XLSX)
