# Supplementary figures and images for: Prognostic Significance of Concurrent Hypovascular and Hypervascular Nodules in Patients with Hepatocellular Carcinoma
Source: PLoS One. 2016 Sep 20;11(9):e0163119. doi: 10.1371/journal.pone.0163119 (PMC5029907; doi:10.1371/journal.pone.0163119)

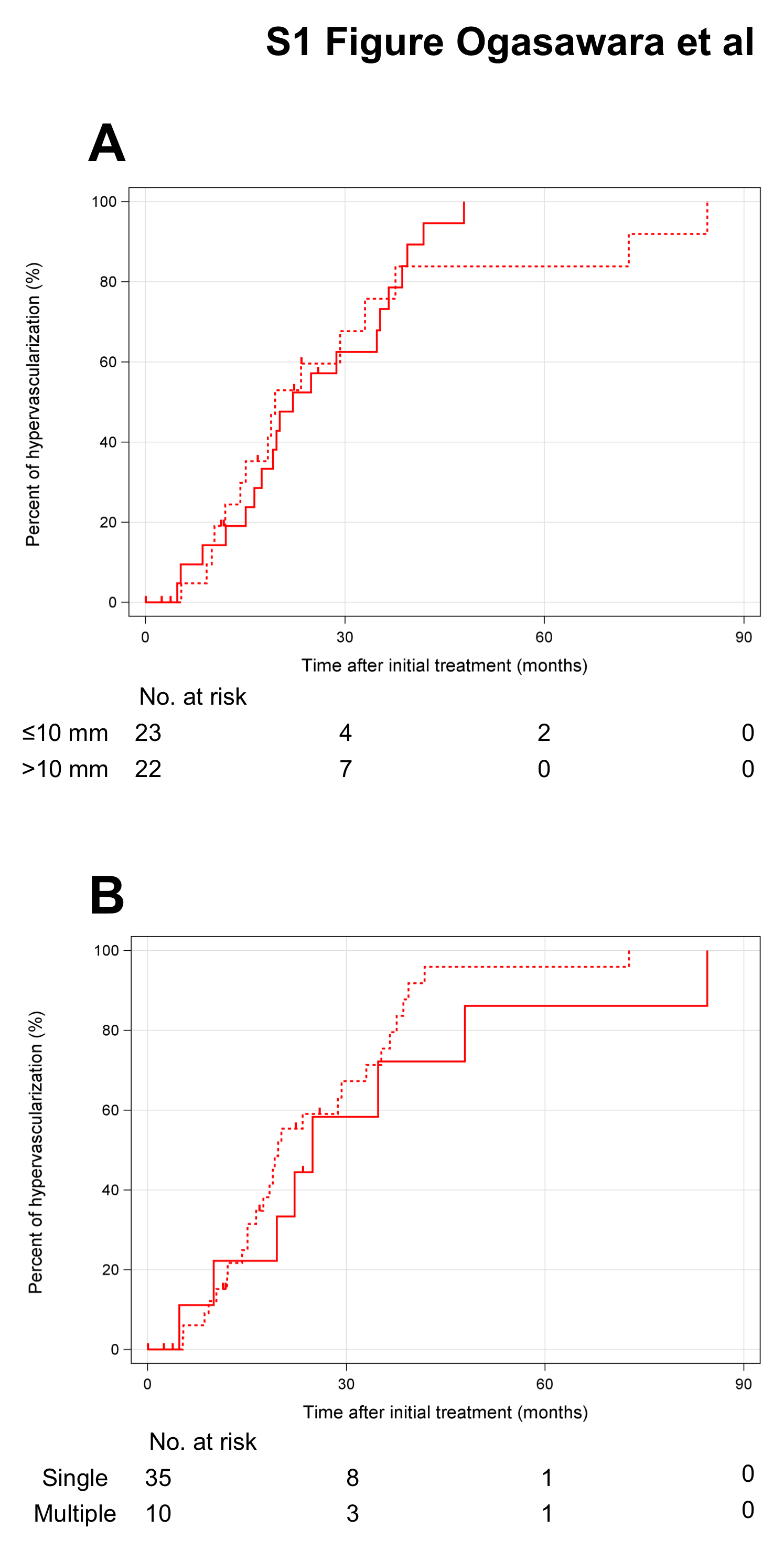

Supplement: S1 Fig — Kaplan-Meier curve of time to hypervascularization for size [A: ≤10 mm (dashed line) vs. > 10 mm (solid line)] and [B: single (dashed line) vs. multiple (solid line)] of hypovascular nodules in patients who had hypovascular nodules. (TIF) [file pone.0163119.s001.tif]

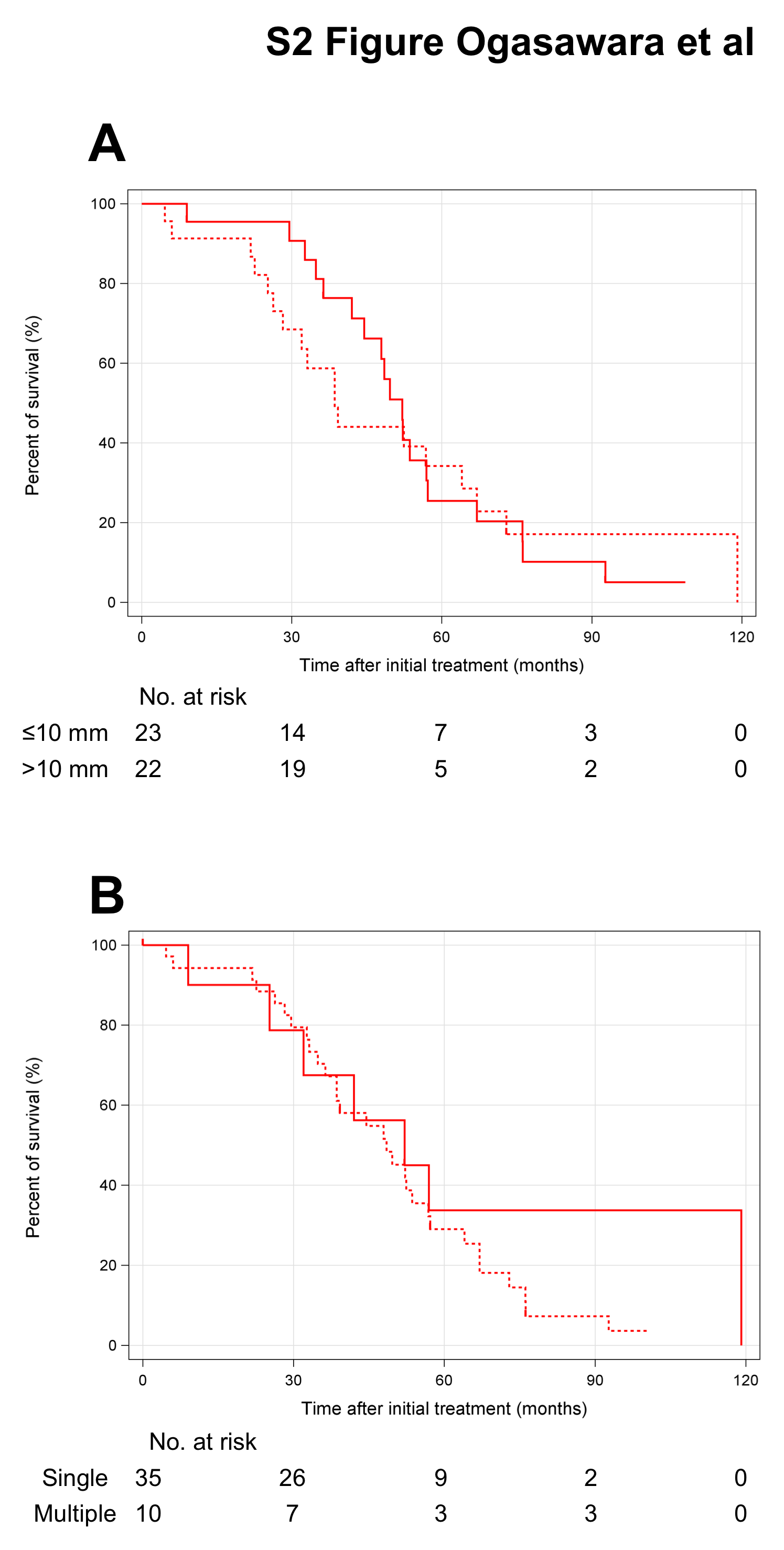

Supplement: S2 Fig — Kaplan-Meier survival curve for size [A: ≤10 mm (dashed line) vs. > 10 mm (solid line)] and [B: single (dashed line) vs. multiple (solid line)] of hypovascular nodules in patients who had hypovascular nodules. (TIF) [file pone.0163119.s002.tif]
