# Supplementary material for: Prognostic Significance of Concurrent Hypovascular and Hypervascular Nodules in Patients with Hepatocellular Carcinoma
Source: PLoS One. 2016 Sep 20;11(9):e0163119. doi: 10.1371/journal.pone.0163119 (PMC5029907; doi:10.1371/journal.pone.0163119)
Supplement: S1 Table — (PDF) [file pone.0163119.s003.pdf]

S1 Table. Characteristics of initial recurrence

	**Hypervascular HCC only (Group A)**	**Hypovascular nodules co-existing with hypervascular HCC (Group B)**	***P***
**Number of recurrences**	72	36	
**Stage at the time of initial recurrence**, n (%)			
Within ≤ 3 hypervascular HCC lesions that were ≤ 30 mm	47 (65)	25 (69)	0.829
Beyond ≤ 3 hypervascular HCC lesions that were ≤ 30 mm	25 (35)	11 (31)	
**Child-Pugh class at the time of initial recurrence**, n (%)			
A	55 (76)	22 (61)	0.180
B	16 (22)	12 (33)	
C	1 (1)	2 (6)	
**Treatment of initial recurrence**, n (%)			
Local ablation	39 (54)	19 (53)	0.951
TACE	24 (33)	13 (36)	
Others	9 (13)	4 (11)	

Abbreviations: HCC, hepatocellular carcinoma; TACE, transcatheter arterial chemoembolization
