# Supplementary material for: Prognostic Significance of Concurrent Hypovascular and Hypervascular Nodules in Patients with Hepatocellular Carcinoma
Source: PLoS One. 2016 Sep 20;11(9):e0163119. doi: 10.1371/journal.pone.0163119 (PMC5029907; doi:10.1371/journal.pone.0163119)
Supplement: S2 Table — (PDF) [file pone.0163119.s004.pdf]

S2 Table. Treatment of clinical course of hepatocellular carcinoma

	**Hypervascular HCC only (Group A)**	**Hypovascular nodules co-existing with hypervascular HCC (Group B)**	***P***
**Total number of treatment**, n (%)			
≤ 3	61 (57)	24 (53)	0.722
> 3	46 (43)	21 (47)	
Median (range)	3 (1–9)	3 (1–11)	
**Local ablation (excluding initial treatment)**, n (%)			
Absent	39 (36)	19 (42)	0.584
Present	68 (64)	26 (58)	
**TACE**, n (%)			
Absent	50 (47)	20 (44)	0.859
Present	57 (53)	25 (56)	
**Others**, n (%)			
Absent	83 (78)	39 (83)	0.265
Present	24 (22)	6 (17)	
**Cause of death**, n (%)			
HCC	28 (26)	14 (31)	0.555
Liver dysfunction	13 (12)	10 (22)	0.138
Others	18 (17)	14 (31)	0.080

Abbreviations: HCC, hepatocellular carcinoma; TACE, transcatheter arterial chemoembolization
