# Supplementary material for: Prognostic Significance of Concurrent Hypovascular and Hypervascular Nodules in Patients with Hepatocellular Carcinoma
Source: PLoS One. 2016 Sep 20;11(9):e0163119. doi: 10.1371/journal.pone.0163119 (PMC5029907; doi:10.1371/journal.pone.0163119)
Supplement: S3 Table — (PDF) [file pone.0163119.s005.pdf]

S3 Table. Univariate analysis based on the contribution of hypovascular nodule prognosis

Variables	Univariate analysis		*P*
	Hazard ratio	95% CI	
**Gender, male**			
Absent	Reference		
Present	1.286	0.802–2.062	0.296
**Age, > 67 years**			
Absent	Reference		
Present	1.444	0.964–2.163	0.075
**HBs-Ag positive**			
Absent	Reference		
Present (treated by NA)	1.187	0.547–2.576	0.665
Present	0.180	0.024–1.337	0.094
**HCV-Ab positive**			
Absent	Reference		
Present	1.637	0.908–2.952	0.101
**Child–Pugh B**			
Absent	Reference		
Present	1.770	1.091–2.873	0.021
**Multiple or > 20-mm hypervascular HCC**			
Absent	Reference		
Present	1.392	0.924–2.098	0.114
**AFP, > 100 ng/mL**			
Absent	Reference		
Present	1.006	0.600–1.685	0.983
**Initial local therapy, PEI**			
RFA	Reference		
PEI	1.246	0.830–1.872	0.288
**Hypovasulcar nodules**			
Absent	Reference		
Present	2.072	1.364–3.148	0.001

Abbreviations: HBs-Ag, hepatitis B surface antigen; NA, nucleos(t)ide analogue; HCV-Ab, hepatitis C virus antibody; HCC, hepatocellular carcinoma; AFP, alpha-fetoprotein; RFA, radiofrequency ablation; PEI, percutaneous ethanol injection
